# The efficacy and safety of ciprofol use for the induction of general anesthesia in patients undergoing gynecological surgery: a prospective randomized controlled study

**DOI:** 10.1186/s12871-022-01782-7

**Published:** 2022-08-03

**Authors:** Ben-zhen Chen, Xin-yu Yin, Li-hua Jiang, Jin-hui Liu, Yan-yan Shi, Bi-ying Yuan

**Affiliations:** 1 Department of Anesthesiology, Sichuan Provincial Women’s and Children’s Hospital, the Affiliated Women’s and Children’s Hospital of Chengdu Medical College, No. 290, Sha Yan Cun Xi Er Jie, Chengdu, 610045 Sichuan China; 2 Department of Operating Room Nursing, Sichuan Provincial People’s Hospital, University of Electronic Science and Technology of China, Chengdu, 610072 China; 3 Department of Laboratory Medicine, Sichuan Provincial Women’s and Children’s Hospital, the Affiliated Women’s and Children’s Hospital of Chengdu Medical College, Chengdu, 610045 Sichuan China

**Keywords:** Ciprofol, General anesthesia, Induction, Gynecological surgery

## Abstract

**Background:**

Ciprofol is a recently developed, short-acting γ-aminobutyric acid receptor agonist sedative that is more potent than propofol, but there have been few clinical studies of this agent to date. Here, we sought to examine the safety and efficacy of ciprofol use for the induction of general anesthesia in individuals undergoing gynecological surgery.

**Methods:**

Women between the ages of 18 and 60 years (ASA physical status 1 or 2) who were scheduled to undergo elective gynecological surgery under general anesthesia were randomly assigned to two equally sized groups in which anesthesia induction was performed using either ciprofol or propofol. General anesthesia induction success rates were the primary outcome for this study, while secondary outcomes included changes in BIS during the 10 min following the first administration of the study drug, the duration of successful induction, and adverse event incidence.

**Results:**

A total of 120 women were included in the study. A 100% rate of successful induction was achieved in both the ciprofol and propofol groups, with no significant differences between these groups with respect to the duration of successful induction (34.8 ± 15.5 s vs 35.4 ± 9.5 s, *P* = 0.832), the time to the disappearance of the eyelash reflex (33.7 ± 10.6 s vs 34.0 ± 6.5 s, *P* = 0.860), or tracheal intubation (58.2 ± 31.1 s vs 53.9 ± 25.4 s, *P* = 0.448). Adverse event rates, including intubation responses, were significantly lower in the ciprofol group as compared to the propofol group(20% vs 48.33%, *P* = 0.0019). Ciprofol was associated with reduced injection pain relative to propofol (16.7% vs 58.3%, *P* < 0.001).

**Conclusions:**

Ciprofol exhibits comparable efficacy to that of propofol when used for the induction of general anesthesia in individuals undergoing gynecological surgery and is associated with fewer adverse events.

**Supplementary Information:**

The online version contains supplementary material available at 10.1186/s12871-022-01782-7.

## Background

The sedative-hypnotic agent propofol is frequently used for the induction and maintenance of general anesthesia, and it is also utilized for monitored anesthesia care (MAC) sedation owing to its rapid onset of activity, short-acting nature, and subsequent rapid awakening in treated patients [1–4]. However, propofol also inhibits the circulatory and respiratory systems and is associated with other adverse events that can limit its clinical application [5–7]. There is thus a need for clinical anesthesiologists to be able to select alternative drugs that maximize patient safety and comfort without compromising efficacy in the context of anesthesia induction. Ciprofol is a recently developed, short-acting γ-aminobutyric acid (GABA) receptor agonist that serves as an anesthetic and sedative, and is more potent than propofol [8, 9]. It has shown promise for use in the intravenous induction of general anesthesia and the sedation of patients, with multiple preclinical and clinical studies having indicated that ciprofol exhibits dose-related sedative-hypnotic effects, rapid onset, rapid offset, a potency 4–6 times greater than propofol, and minor residual side effects following the administration of a single therapeutic dose. In general, ciprofol is associated with similar adverse side effects to those observed following propofol treatment, primarily affecting the respiratory and cardiovascular systems. There is also some evidence to suggest that ciprofol may exhibit lower rates of injection site pain and adverse respiratory effects as compared to propofol given that it is prepared at a lower concentration in the aqueous phase of the emulsion [10]. However, as ciprofol was only recently developed, limited data are currently available regarding its use for the induction of general anesthesia. As such, we herein designed and executed a prospective, randomized, double-blind study exploring the safety and efficacy of ciprofol when used for general anesthesia induction in individuals undergoing gynecological surgery, with propofol serving as a control.

## Methods

### Research ethics

The present prospective, double-blind, single-center study was conducted at Sichuan Provincial Women’s and Children’s Hospital. The Medical Ethics Committee of Sichuan Provincial Women’s and Children’s Hospital approved the present study (review board number: 20201113–114), which was registered in the Chinese Clinical Trial Registry in 08/04/2021(Registration number: ChiCTR2100045211). Written informed consent was obtained from all participants. This manuscript adheres to the applicable EQUATOR guidelines.

### Study design

**Fig. 1:**
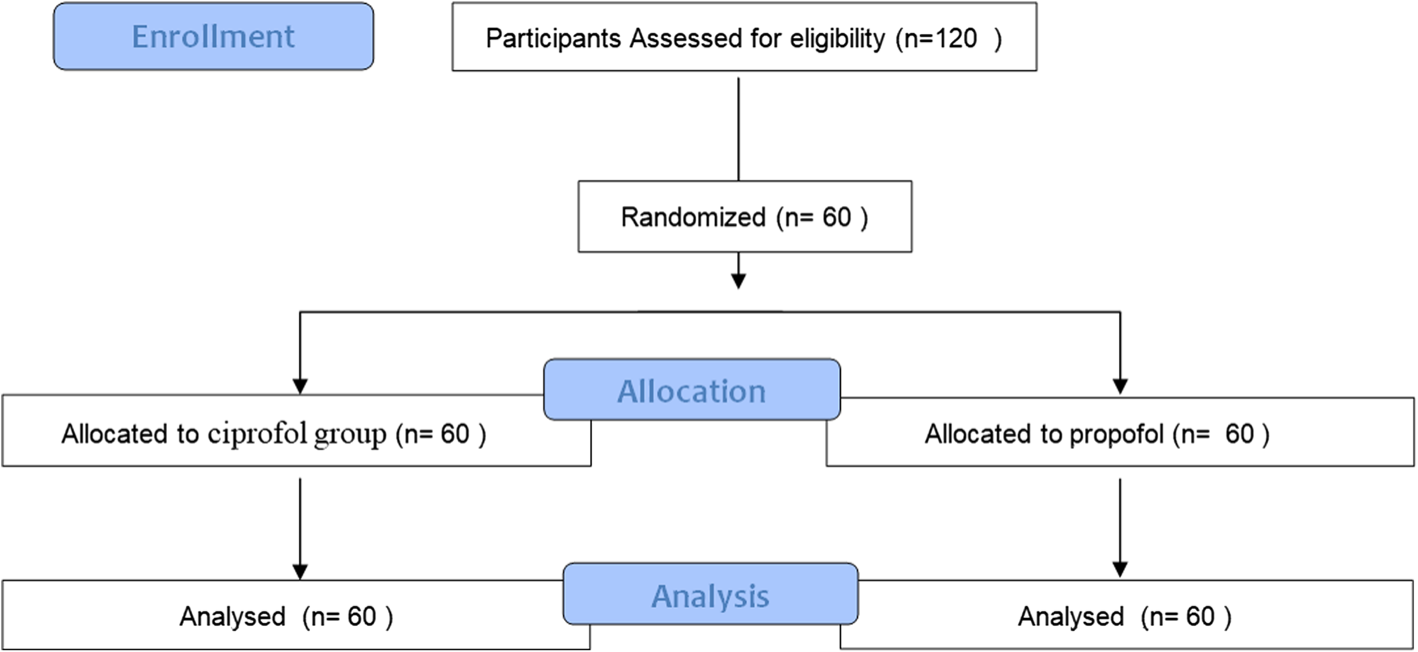
CONSORT Flow Diagram

This study was a non-inferiority design, and the primary endpoint was the success rate of induction of general anesthesia. In type I error (false positive), 0.025 (unilateral), Power of test is 80%. The success rate of general anesthesia induction for both cyprofol and propofol positive control drugs was set to be 98%, the non-inferiority margin was defined as 8%, and a total of 120 hospitalized subjects undergoing elective surgery in our hospital from June 2021 to March 2022 were included in this study. They were equally assigned to the ciprofol group and the propofol group, with 60 subjects in each group. Consecutive adult females between the ages of 18 and 60 (ASA physical status: I or II) who were scheduled to undergo elective gynecological surgery under general anesthesia were recruited for the present study. The CONSORT Flow Diagram was listed in Fig. 1. Patients were excluded if they suffered from morbid obesity, egg/soy allergies, diabetes mellitus, gastroesophageal reflux, or symptomatic neurological, respiratory, or cardiovascular disease in this study. In addition, women who were pregnant, lactating, or planning to become pregnant within 1 month after the trial were excluded. After assessing patient eligibility, they were informed regarding the study by members of the study team, with written informed consent then being obtained.

### Randomisation, blinding, and general anesthesia induction

Patients were randomly assigned to two equally sized groups using a computer-generated random number table, with this list being maintained in a locked cabinet to which only nursing staff members without any direct involvement in patient care or the study as a whole had access. These nurses prepared study medications in a closes room without observation based on patient group assignments.

Patients in this study were fasted for a minimum of 6 h without premedication. Following arrival in the operating room, patients were monitored via electrocardiography, respiratory rate, pulse oximetry, bispectral index (BIS), and continuous noninvasive arterial blood pressure (CNAP). An 18-Gauge intravenous cannula was inserted into a vein in the dorsum of the right hand.

Intravenous midazolam (0.03 mg/kg) and sufentanil (0.3 μg/kg) were used to start general anesthesia induction, followed 2 min later by the manual injection for 30 s of ciprofol (0.4 mg/kg; Haisco Pharmaceutical Group Co. Ltd, China) or medium-and long-chain triglyceride (MCT/LCT) propofol (2 mg/kg; Fresenius Kabi Deutschland GMBH) as appropriate. Patients started receiving preoxygenation (breath spontaneous using a closed mask with 100% oxygen) after intravenous midazolam and sufentanil being administrated. When spontaneous breathing disappeared, it switched to manual controlled breathing. The patient's respiratory rate remained between 12 and 20 during induction before the administration of rocuronium and the SPO2 remained 100%. Patient responses were then monitored until there was clinical evidence that anesthetization was effective. Sedation levels in subjects were assessed using the Modified Observer’s Assessment of Alertness/Sedation (MOAA/S) scale [11–13]. The patients’ baseline MOAA/S were measured before administering midazolam. Time to eyelash reflex disappearance from the beginning of study drug administration were assessed every 5 s through touch the eyelashes with a sterile cottom swab gently. The following two conditions should be met for successful induction of anesthesia: 1) MOAA/S ≤ 1; 2) No alternative hypnotics were used. If successful induction was not achieved within 1 min, the addition of an additional dose of ciprofol (0.2 mg/kg) or MCT/LCT propofol (1 mg/kg) was allowed for patients in the corresponding treatment groups. When induction remained unsuccessful following the administration of two additional study drug doses, propofol was administered to complete the induction. Once induction was successful, rocuronium (0.6 mg/kg) was administered, and endotracheal intubation was conducted after the muscle relaxant took effect, the intubation time point was uniformly defined as 4 min after the beginning of induction(administration of study drug). 2 min after intubation, anesthesia was maintained with sevoflurane in oxygen 50% and/or other intravenous anesthetics depending on the need for surgery or the personal habits of the anesthesiologist.

### Outcomes

The present study was conducted to assess the safety and efficacy of ciprofol. Evaluation was performed every 30 s after administering midazolam, and the evaluation interval was shortened to 5 s at the beginning of study drug administration until the MOAA/S score was ≤ 1, and the longest evaluation time was no more than 3 min after the beginning of study drug administration. The success rate of general anesthesia induction was the primary outcome for the present study, and was defined as the percentage of successful induction cases in each group(the lack of any need for an alternative sedative/anesthetic drug or the need for > 2 top-up study drug doses following the start of study drug administration).

Secondary study outcomes included: (1) the time to onset of successful induction from the initiation of study drug treatment to a MOAA/S score ≤ 1; (2) the incidence of injection site pain as detected by a withdrawal response or a numeric rating scale value ≥ 3, Subjects were asked “Do you feel pain in the arm where the drug was injected?” during the injection. If the answer was “yes”, Subjects were asked to describe the intensity of the pain (0 to10 points indicated “no pain” to “unbearable pain”). Evaluation was performed at least once during the study drug injection until the successful induction of the subjects (MOAA/S ≤ 1); (3) time to eyelash reflex disappearance from the beginning of study drug administration; (4) changes in the bispectral index (BIS) during the 10-min interval following the start of study drug administration; and (5) the utilization of study drug top-up doses and/or rescue/remediation drugs.

### Statistical analysis

SPSS v26.0 (SPSS, Inc., IL, USA) was used for all statistical analyses. Data were compared between groups using unpaired t-tests, chi-squared tests, Fisher’s exact test, or Kruskal–Wallis one-way ANOVAs with Dunn’s multiple comparison test as appropriate. *P* < 0.05 was the significance threshold.

## Results

From April 2021 – November 2021, A total of 120 women were included in the study. All enrolled patients successfully completed this trial. There were no significant differences between the propofol and ciprofol groups with respect to patient age, baseline weight, BMI, ASA physical status, or operative duration (Table 1). The induction success rates were 100% in both groups. No significant differences were observed between these groups with respect to the onset of successful induction (34.8 ± 15.5 s vs 35.4 ± 9.5 s, *P* = 0.832), or the time to eyelash reflex disappearance (33.7 ± 10.6 s vs 34.0 ± 6.5 s, *P* = 0.860) (Table 2).

**Table 1:** Participant baseline characteristics

Patient characteristics	Ciprofol group (*n* = 60)	Propofol group (*n* = 60)	*P*-value
Age,year, mean ± SDs	33.9 ± 9.1	33.8 ± 9.6	0.626
Height, cm, mean ± SDs	159.3 ± 3.8	158.5 ± 5.2	0.915
Weight, kg, mean ± SDs	56.9 ± 7.9	54.0 ± 9.1	0.991
BMI, kg/m^2^, mean ± SDs	22.2 ± 3.2	21.4 ± 2.8	0.909
ASA status, n(%)			
I	32(53.3%)	34(56.7%)	0.732
II	28(46.7%)	26(43.3%)	0.793
Operative duration, min, mean ± SDs	55.2 ± 20.5	51.4 ± 23.1	0.645

*BMI* Body mass index, *ASA* American Society of Anesthesiologists

**Table 2:** Key study outcomes

Variable	Ciprofol group (*n* = 60)	Propofol group (*n* = 60)	*P* value
Onset of successful induction (s, mean ± SD)	34.8 ± 15.5	35.4 ± 9.5	0.832
Time to disappearance of eyelash reflex (s, mean ± SD)	33.7 ± 10.6	34.0 ± 6.5	0.860

Successful induction: no need for alternative sedatives or anesthesia drugs, and no need for > 2 study drug top-up doses within 5 min following the start of study drug administration; Time to the disappearance of eyelash reflex: the interval between the initiation of study drug administration and eyelash reflex disappearance

**Fig. 2:**
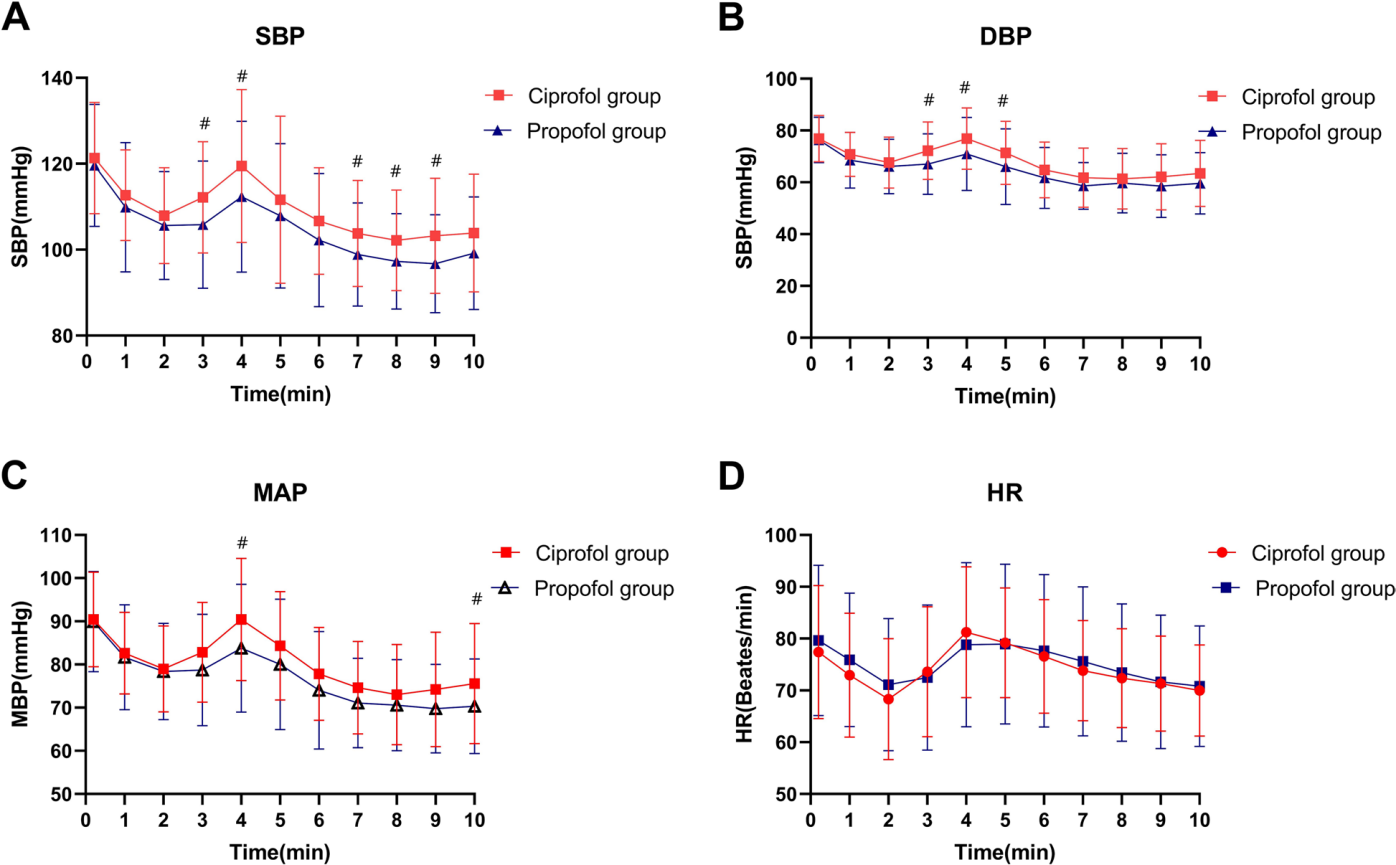
Changes in blood pressure and heart rate following anesthesia induction; Time 0 was defined as the baseline value 10 s prior to the administration of the study drug; **#**: *P* < 0.01

**Fig. 3:**
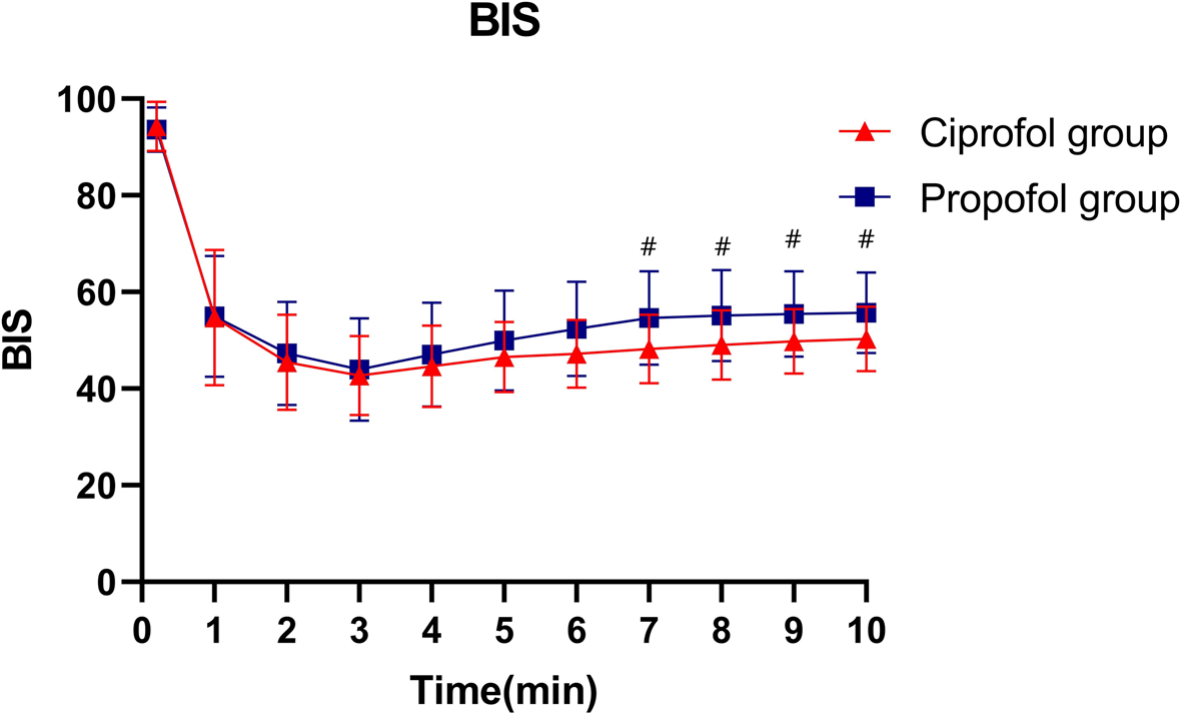
Changes in the bispectral index (BIS) following anesthesia induction; Time 0 was defined as the baseline value 10 s prior to the administration of the study drug; **#**: *P* < 0.01

Blood pressure values declined significantly within 2 min following study drug administration in both groups before gradually returning to baseline following endotracheal intubation. Within the initial 10 min following study drug administration, blood pressure values declined significantly less than in the propofol group (Fig. 2A, B, C, *P* < 0.05). Comparable changes in heart rate were observed in both groups (Fig. 2D). The changes of BIS following anesthesia induction of the two groups were shown in Fig. 3. The mean range of BIS in ciprofol group was significantly lower than that in the propofol group(13.82 ± 8.10 Vs 18.53 ± 9.62, *P* < 0.01).

The adverse events experienced proximal to anesthesia induction among patients in the present study are compiled in Table 3. Overall rates of adverse events including intubation responses were reduced in the ciprofol group relative to the propofol group (20% vs 48.33%, *P* = 0.0019). Injection pain incidence was also significantly reduced in the ciprofol group relative to the propofol group (16.7% vs 58.3%, *P* < 0.001). Overall, the only adverse events that occurred among study patients were mild, with no evidence of severe adverse events or other notable abnormal outcomes.

**Table 3:** Adverse event incidence during the peri-anesthetization period

Variable	Ciprofol group (*n* = 60)	Propofol group (*n* = 60)	*P*-value
Intubation response, n(%)			
swallowing,	0	3(5%)	
cough	0	1(1.67%)	
body moving	0	2(3.33%)	
tears	0	1(1.67%)	
hypertension	4(6.67%)	2(3.33%)	
hypotension	5(8.33%)	16(26.67%)	
bradycardia	2(3.33%)	1(1.67%)	
tachycardia	1(1.67%)	3(5%)	
total	12(20%)	29(48.33%)	0.0019
Injection-site pain, n(%)	10(16.7%)	35(58.3%)	< 0.001

Hypertension, hypotension, bradycardia and tachycardia were defined as occurred within 10 min after administration of the study drug were recorded; hypertension: an increase of 20% than baseline value; hypotension: an decrease of 20% than baseline; bradycardia: heart rates less than 55 beats/min; tachycardia: heart rates more than 100 beats/min; Injection-site pain: with a withdrawal response or a numeric rating scale value ≥ 3

## Discussion

Here, we examined the relative safety and efficacy of ciprofol when used for general anesthesia induction in individuals undergoing elective gynecological surgery. These analyses revealed ciprofol to be comparable to propofol with respect to general anesthesia induction while being associated with lower rates of adverse events including intubation responses in comparison to the latter sedative drug. Moreover, injection site pain was less common among patients treated with ciprofol relative to those treated with propofol. The mean BIS range in the ciprofol groups was significantly lower than that in the propofol groups, suggesting ciprofol induction to be associated with more stable BIS changes. This study is the first to have specifically analyzed the safety and efficacy of utilizing ciprofol for the induction of general anesthesia in gynecological surgery patients. Key study efficacy outcomes included the duration of successful induction, the time to eyelash reflex disappearance, and the time time to tracheal intubation, with all of these parameters being comparable in the ciprofol (0.4 mg/kg) and propofol (2 mg/kg) groups. Our data also suggest that ciprofol was associated with slightly less pronounced effects on the cardiovascular system, with its impacts on heart rate and blood pressure being non-inferior to those of propofol. Ciprofol was also significantly safer than propofol as indicated by the reduced incidence of intubation responses in this patient group, in line with prior preclinical work and findings from preliminary clinical efficacy studies [9, 10, 14].

Ciprofol is a recently developed intravenous anesthetic drug chat is structurally similar to propofol and exhibits desirable pharmacodynamic properties including both rapid onset and rapid offset [8]. Moreover, it binds to the γ-aminobutyric acid type A (GABA_A_) receptor more tightly than does propofol and exhibits reduced lipophilicity and a more suitable steric bulk. Injection pain is among the most frequently reported adverse reactions associated with propofol administration, resulting in tension, anxiety, discomfort, and body movements with the potential to impact hemodynamic stability during induction [6, 15]. Propofol-related injection pain has been estimated to occur in 50–80% of cases [16–18]. In contrast, our results indicated that ciprofol was associated with lower rates of injection pain as compared to propofol (16.7% *vs* 58.3%). This difference is likely attributable to the differences in the concentrations of these two drugs in the aqueous phase of the injection solution, with the higher concentration of propofol contributing to greater pain on injection [19]. Consistent with this hypothesis, prior studies have reported lower rates of injection site pain when the propofol concentration in the aqueous phase of the emulsion was reduced [20–22]. Here, we found ciprofol-related injection site pain to be mild, likely owing to the fact that ciprofol was formulated as an oil-in-water emulsion due to its poor aqueous solubility [23]. The greater hydrophobicity and lower plasma concentrations of ciprofol relative to propofol may also be linked to the lower rates of injection pain [14]. Given the limitations and shortcomings of propofol currently used in the anesthetics market, we can predict that ciprofol has the potential to become an alternative intravenous anesthetic after medium and long chain triglyceride (MCT/LCT) propofol. Ciprofol, with its unique advantages, can better serve clinical practice, and benefit patients.

There are some limitations in this study. Only the subjects of ASA physical status 1 or 2 were included. Subjects with respiratory or cardiovascular disease were excluded. The efficacy and safety of maintenance period were not investigated. Further studies should be conducted to explore the efficacy and safety of ciprofol in induction and maintenance of anesthesia in patients with respiratory or cardiopulmonary disease.

Overall, the results of this study indicate that ciprofol is as effective as propofol when used for general anesthesia induction in individuals undergoing gynecological surgery while being associated with lower rates of adverse events.

## Supplementary Information

**Additional file 1.** Modified Observer’s Assessment of Alertness/Sedation (MOAA/S) Scale.

## Data Availability

The data that support the findings of this study are available from the corresponding author, Benzhen Chen, upon reasonable request.
