# Supplementary material for: The efficacy and safety of ciprofol use for the induction of general anesthesia in patients undergoing gynecological surgery: a prospective randomized controlled study
Source: BMC Anesthesiol. 2022 Aug 3;22:245. doi: 10.1186/s12871-022-01782-7 (PMC9347095; doi:10.1186/s12871-022-01782-7)
Supplement: Supplementary file 1 — Additional file 1. Modified Observer’s Assessment of Alertness/Sedation (MOAA/S) Scale. [file 12871_2022_1782_MOESM1_ESM.docx]

Modified Observer’s Assessment of Alertness/Sedation (MOAA/S) Scale

| Score | Response | ASA Classification |
| --- | --- | --- |
| 5  4  3  2  1  0 | Responds readily to name spoken in a normal tone  Lethargic response to name spoken in a normal tone  Responds only after name is called loudly or repeatedly  Responds only after mild prodding or shaking  Responds only after painful trapezius squeeze  Does not respond to painful trapezius squeeze | Minimum  Moderate  Moderate  Moderate  Deep  Deep/general anesthesia |

ASA: American Society of Anesthesiologists.
